# Induction of the Wnt Antagonist Dickkopf-1 Is Involved in Stress-Induced Hippocampal Damage

**DOI:** 10.1371/journal.pone.0016447

**Published:** 2011-01-27

**Authors:** Francesco Matrisciano, Carla L. Busceti, Domenico Bucci, Rosamaria Orlando, Alessandra Caruso, Gemma Molinaro, Irene Cappuccio, Barbara Riozzi, Roberto Gradini, Marta Motolese, Filippo Caraci, Agata Copani, Sergio Scaccianoce, Daniela Melchiorri, Valeria Bruno, Giuseppe Battaglia, Ferdinando Nicoletti

**Affiliations:** 1 Department of Physiology and Pharmacology, University “Sapienza”, Roma, Italy; 2 Istituto Neurologico Mediterraneo Neuromed, Pozzilli, Italy; 3 Istituto Casimiro Mondino, Pavia, Italy; 4 Department of Experimental Medicine, University “Sapienza”, Roma, Italy; 5 Department of Pharmaceutical Sciences, University of Catania, Catania, Italy; 6 Istituto San Raffaele Pisana, Roma, Italy; Federal University of Rio de Janeiro, Brazil

## Abstract

The identification of mechanisms that mediate stress-induced hippocampal damage may shed new light into the pathophysiology of depressive disorders and provide new targets for therapeutic intervention. We focused on the secreted glycoprotein Dickkopf-1 (Dkk-1), an inhibitor of the canonical Wnt pathway, involved in neurodegeneration. Mice exposed to mild restraint stress showed increased hippocampal levels of Dkk-1 and reduced expression of β-catenin, an intracellular protein positively regulated by the canonical Wnt signalling pathway. In adrenalectomized mice, Dkk-1 was induced by corticosterone injection, but not by exposure to stress. Corticosterone also induced Dkk-1 in mouse organotypic hippocampal cultures and primary cultures of hippocampal neurons and, at least in the latter model, the action of corticosterone was reversed by the type-2 glucocorticoid receptor antagonist mifepristone. To examine whether induction of Dkk-1 was causally related to stress-induced hippocampal damage, we used *doubleridge* mice, which are characterized by a defective induction of Dkk-1. As compared to control mice, *doubleridge* mice showed a paradoxical increase in basal hippocampal Dkk-1 levels, but no Dkk-1 induction in response to stress. In contrast, stress reduced Dkk-1 levels in *doubleridge* mice. In control mice, chronic stress induced a reduction in hippocampal volume associated with neuronal loss and dendritic atrophy in the CA1 region, and a reduced neurogenesis in the dentate gyrus. *Doubleridge* mice were resistant to the detrimental effect of chronic stress and, instead, responded to stress with increases in dendritic arborisation and neurogenesis. Thus, the outcome of chronic stress was tightly related to changes in Dkk-1 expression in the hippocampus. These data indicate that induction of Dkk-1 is causally related to stress-induced hippocampal damage and provide the first evidence that Dkk-1 expression is regulated by corticosteroids in the central nervous system. Drugs that rescue the canonical Wnt pathway may attenuate hippocampal damage in major depression and other stress-related disorders.

## Introduction

Abnormalities in mechanisms of resilience to stress are implicated in the pathophysiology of major depressive disorders [1]. Severe depression or depression associated with psychotic symptoms is associated with hypercortisolemia [2], which reflects an impaired negative feed-back of glucocorticoids on the activity of the hypothalamic-pituitary-adrenal (HPA) axis [3], an enhanced adrenal response to adrenocorticotropic hormone (ACTH) [4], or other mechanisms. Excess of glucocorticoids triggers a series of pathological events that may be involved in the pathophysiology of hippocampal atrophy in depressed patients [5]–[7]. Chronic stress or exogenous glucocorticoids reduce dendritic arborisation in hippocampal neurons [8]–[14] and can also cause hippocampal neuronal death [15]–[17]. In addition, chronic stress reduces neurogenesis in the hippocampal dentate gyrus [1], an event that further links HPA hyperactivity to hippocampal atrophy. Understanding the mechanisms that underlie hippocampal damage in response to stress/glucocorticoids may shed new lights into the pathophysiology of mood disorders and stress-related cognitive dysfunctions, and may lead to the identification of new therapeutic targets. Glucocorticoids are toxic to hippocampal neurons *via* the activation of low-affinity glucocorticoid receptors (GRs), which triggers apoptotic death [18], and increases neuronal vulnerability to excitotoxins, reactive oxygen species and other insults [16], [15], [19]–[24]. However, the molecular events that mediate neuronal death in response to stress/glucocorticoids are only partially identified. We have focused on the canonical Wnt pathway, which has an established role in developmental processes, but has recently been implicated in mechanisms of neurodegeneration/neuroprotection in the adult brain [25]–[27]. In addition, Wnt signalling regulates the fate of adult hippocampal neural stem/progenitor cells [28], [29]. The canonical Wnt pathway controls the stability of the intracellular protein, β-catenin, which, if no degraded, translocates to the nucleus, binds to lymphoid enhancer-binding factor (LEF) and T cell factor (TCF) proteins, and acts as a transcriptional co-activator to regulate the expression of Wnt-dependent genes. In the absence of Wnt ligands, β-catenin is phosphorylated by a “degradation complex” that comprises glycogen synthase kinase 3β (GSK3β), casein kinase 1α, Axin, and adenomatous polyposis coli (APC). Phosphorylation of β-catenin leads to its ubiquitynation by a specific E3 ligase, and proteasomal degradation. Interaction of Wnt glycoproteins with 7-TM *Frizzled* receptors and low density lipoprotein receptor-related proteins 5/6 (LRP5/6) co-receptors inhibits the β-catenin degradation complex *via* the Dishevelled cytoplasmic phosphoproteins, thus enhancing the intracellular levels of β-catenin [30]. The canonical Wnt pathway is inhibited by Dickkopf-1 (Dkk-1), a member of the Dkk family of secreted Wnt antagonists [31]. Dkk-1 binds to LRP5/6 and the single-pass transmembrane proteins Kremen-1 and -2 forming a ternary complex that disrupts Wnt signalling by promoting endocytosis of LRP5/6 [32]. Induction of Dkk-1 with the ensuing inhibition of the canonical Wnt pathway has been linked to processes of neuronal death in cultures challenged with excitotoxins or β-amyloid, and in animal models of global and focal brain ischemia or temporal lobe epilepsy. In addition, expression of Dkk-1 is increased in degenerating neurons of the Alzheimer's brain and in hippocampal neurons of patients with mesial temporal lobe epilepsy associated with Ammon's horn sclerosis [33]–[37]. We now show that (i) acute and chronic mild restraint stress selectively enhances Dkk-1 expression in the hippocampus; (ii) the action of stress requires the integrity of the HPA axis and is mimicked by exogenous corticosterone; and (iii) mice with hypomorphic Dkk-1 alleles are protected against stress-induced hippocampal damage.

## Results

### 1. Induction of Dkk-1 in the hippocampus of mice subjected to mild restraint stress

**Figure 1:**
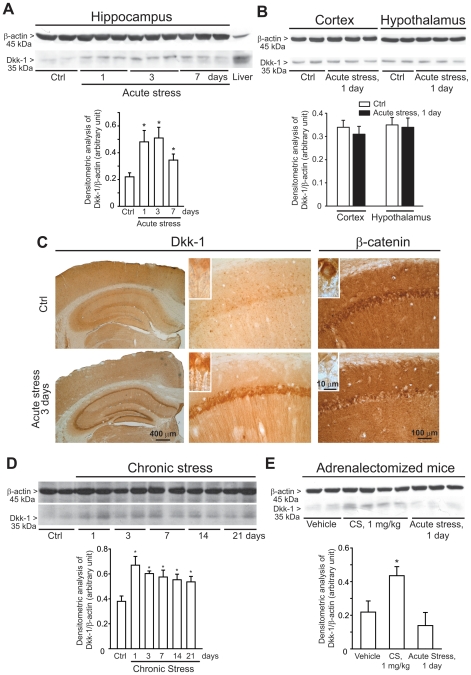
Induction of Dkk-1 in the hippocampus of CD1 mice exposed to acute or chronic restraint stress. A representative immunoblot of Dkk-1 in hippocampal extracts of mice at 1, 3 or 7 days following a single exposure to mild restrain stress (“acute stress”) is shown in (A). Data are means ± S.E.M. of 6–9 individual determinations. *p<0.05 (One-way ANOVA + Fisher's PLSD vs. Control (Ctrl) mice. A representative immunoblot of Dkk-1 in extracts from cortex and hypothalamus of mice at 1 day following a single exposure to mild restrain stress (“acute stress”) is shown in (B). Data are means ± S.E.M. of 5-6 individual determinations. Immunohistochemical analysis of Dkk-1 and â-catenin in control mice and mice killed 3 days after acute stress is shown in (C). Note the lack of Dkk-1 immunostaining in cell nuclei in the inset at high magnification. Immunoblot analysis of Dkk-1 in mice exposed to repeated episodes of mild restrain stress (once a day for 21 consecutive days) (“chronic stress”) is shown in (D). Mice were killed 24 h after the last exposure to stress at day 1, 3, 7, 14, and 21 (i.e. mice were killed at day 2, 4, 8, 15, and 22). Values are means ± S.E.M. of 6–9 determinations. *p<0.05 (One-way ANOVA + Fisher's PLSD) vs. controls. Immunoblot analysis of Dkk-1 levels in the hippocampus of adrenalectomized mice injected with vehicle or corticosterone (CS, 1 mg/kg, s.c.) or exposed to acute stress is shown in E. The group of adrenalictomized mice was also injected with vehicle to allow comparisons with the two previous groups. Mice were killed 24 h after treatments. Values are means ± S.E.M. of 6–7 determinations. *p<0.05 (One-way ANOVA + Fisher's PLSD) vs. unstressed mice injected with vehicle.

CD1 mice were killed 1, 3 or 7 days after a 3-h exposure to mild restraint stress. Immunoblot analysis with anti-Dkk-1 antibodies showed a band corresponding to the molecular size of Dkk-1 (35 kDa), which was prominent in the mouse liver, used as a control tissue. Dkk-1 levels were very low in the hippocampus of control mice, as expected (see representative immunoblot in Figure 1A). A single exposure to stress substantially increased Dkk-1 levels in the hippocampus after 1 and 3 days. The increase was still visible, albeit to a lesser extent, after 7 days (Figure 1A). No changes in Dkk-1 levels were seen in the cerebral cortex and hypothalamus at least at 1 day after stress (Figure 1B). In another set of experiments, mice were exposed daily to restraint stress for 21 consecutive days. This treatment led to a substantial increase in hippocampal Dkk-1 levels, which peaked in the first 3 days, and could still be detected at 7, 14, and 21 days (Figure 1D). Mice killed 3 days following a single exposure to stress were also used for immunohistochemical analysis, which showed a prominent increase in Dkk-1 immunostaining in all hippocampal subfields in response to stress. Dkk-1 immunostaining was restricted to neurons, spared the cell nucleus, and was detected both intra- and extracellularly, as expected for a secreted protein (Figure 1C). Immunohistochemical analysis was also used for the detection of β-catenin, the levels of which are critically regulated by the Wnt pathway (see Introduction and references therein). Consistent with the increase in Dkk-1 levels, acute stress induced a reduction in β-catenin immunoreactivity in the cytoplasmic and nuclear region of hippocampal pyramidal neurons (Figure 1C). This reduction could not be consistently confirmed by immunoblot analysis, perhaps because of the widespread expression of β-catenin in different cell types within the hippocampus (Figure 1C).

### 2. Mild restraint stress fails to increase hippocampal Dkk-1 levels in adrenalectomized mice

To examine whether the activation of the HPA axis is involved in the increase in hippocampal Dkk-1 levels associated with stress, we used CD1 mice subjected to surgical bilateral adrenalectomy, and exposed to acute mild restraint stress 7 days later. In adrenalectmized mice, hippocampal Dkk-1 levels were nearly undetectable under control conditions, and failed to increase 1 day following acute restraint stress. However, a significant increase in hippocampal Dkk-1 levels was seen when these mice were treated systemically with corticosterone (1 mg/kg, s.c.), and killed 1 day later (Figure 1E).

### 3. Addition of corticosterone induces Dkk-1 expression in organotypic hippocampal cultures and cultured hippocampal neurons

**Figure 2:**
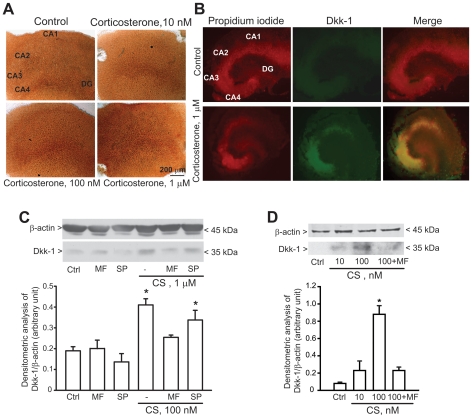
Exogenous corticosterone enhances Dkk-1 expression in organotypical hippocampal slice cultures and cultured hippocampal neurons. Expression of Dkk-1 in organotypic hippocampal cultures exposed to different concentrations of corticosterone for is shown in (A and B). Cultures were incubated with corticosterone or vehicle (Ctrl) for 24 h and then processed for DAB (A) or immunofluorescent (B) analysis. In (B) propidium iodide staining is in red, Dkk-1 immunostaining in green. Note that Dkk-1 induction is visible in CA3, CA4, and CA1 hippocampal subfields. The experiment has been repeated three times with identical results. Induction of Dkk-1 by corticosterone (CS) in primary cultures of mouse or rat hippocampal neurons is shown in C and D, respectively. The time of incubation with CS was 24 h in C and 16 h in D. Note that induction of Dkk-1 by corticosterone was antagonized by mifepristone (MP, 10 µM) but not by spironolactone (SP, 10 µM). MP and SP were applied to the cultures 10 min prior to CS and mainteined in the culture medium for 16–24 h. Ctrl  =  control cultures treated with vehicle. Values are means ± S.E.M. of 5–6 individual determinations from 2 independent cultures. *p<0.05 (One-way ANOVA + Fisher's PLSD) vs. Ctrl.

To examine whether Dkk-1 expression in the hippocampus could be directly induced by glucocorticoids we used two *in vitro* models: (i) organotypic hippocampal cultures; and (ii) primary cultures of hippocampal neurons. In organotypic hippocampal cultures, addition of corticosterone (100 nM and 1 µM) increased Dkk-1 immunoreactivity after 24 h. No increase was seen in response to 10 nM corticosterone (Figure 2A). Immunofluorescent analysis of organotypic hippocampal cultures exposed to 1 µM corticosterone showed that induction of Dkk-1 was prominent in the CA4 and CA3 subfields although it was also visible in the CA1 region and dentate gyrus (Figure 2B). We also used primary cultures of mouse and rat hippocampal neurons at 10 days *in vitro* (DIV). These cultures contain >95% of neurons with 2-3% of contaminating astrocytes (not shown). A single pulse with corticosterone (100 nM) substantially increased Dkk-1 levels in hippocampal neurons, as shown by immunoblot analysis (Figure 2C,D). The action of corticosterone was reversed by the type-2 glucocorticoid receptor (GR) antagonist mifepristone (10 µM), but not by the selective mineralcorticoid receptor (MR) antagonist, spironolactone (10 µM). Neither mifepristone nor spironolactone had any effect on Dkk-1 levels on their own (Figure 2C,D). These data suggest that corticosterone induces Dkk-1 expression in hippocampal neurons *via* the activation of low-affinity type-2 GRs.

### 4. Insertional mutant mice lacking a transcriptional enhancer in the Dkk1 gene (“doubleridge” mice) are resistant to stress-induced hippocampal damage

**Figure 3:**
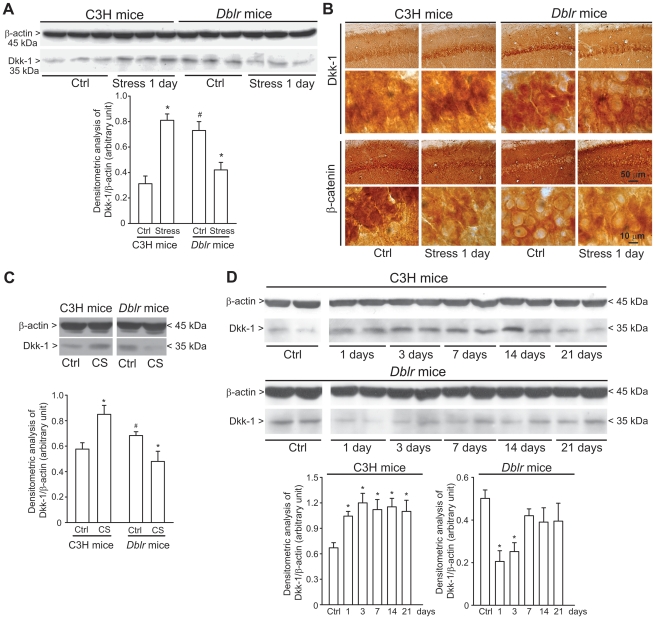
Dkk-1 response to stress or corticosterone in C3H and *doubleridge* mice. Immunoblot analysis of Dkk-1 in the hippocampus of C3H or *doubleridge* (*Dblr*) mice exposed to acute restraint stress and killed 24 h later is shown in (A). Note that basal Dkk-1 levels were higher in the hippocampus of *doubleridge* mice and that acute stress enhanced Dkk-1 levels in C3H control mice but *reduced* Dkk-1 levels in *doubleridge* mice. Values are means ± S.E.M. of 6 determinations. p<0.05 (One-way ANOVA + Fisher's PLSD) vs. the respective control values (*) or vs. unstressed (Ctrl) C3H mice (#). Immunohistochemical analysis of Dkk-1 and β-catenin in the hippocampus of control C3H and *doubleridge* mice and C3H and *doubleridge* mice killed 1 day after acute stress is shown in (B). Immunoblot analysis of Dkk-1 levels in the hippocampus of C3H or *doubleridge* mice subjected to chronic restraint stress (once a day for consecutive 21 days; see legend of Figure 1) is shown in (C). Values are means ± S.E.M. of 5–8 determinations. *p<0.05 (One-way ANOVA + Fisher's PLSD) vs. control mice of the respective strain. In (D), we show the differential effect of 24-h treatment with 1 µM corticosterone (CS) on Dkk-1 levels in primary cultures of hippocampal neurons prepared from C3H or *doubleridge* mice. Values are means ± S.E.M. of 6 determinations from 2 independent cultures. p<0.05 (One-way ANOVA + Fisher's PLSD) vs. the respective control values (*).

We used mice chronically exposed to mild restraint stress to examine whether induction of Dkk-1 with the ensuing inhibition of the canonical Wnt pathway could contribute to stress-induced hippocampal damage. Stress was delivered once a day for 21 days to control C3H mice or to *doubleridge* mice, which show a defective expression of Dkk-1 in response to developmental cues (45), or to neuronal insults in the adult life [37], [46]. A bias inherent to the *doubleridge* model is that these mice show higher basal Dkk-1 protein levels in brain tissue [46], although the Dkk1 gene is not fully responsive to inductive cues. We could not use Dkk1 knockout mice because these mice die before birth [47]. We first examined the Dkk-1 response 1 day after a single exposure to mild restraint stress in *doubleridge* mice and their controls (C3H mice). *Doubleridge* mice showed higher basal Dkk-1 levels in the hippocampus, but they showed an abnormal response to stress. Exposure to stress enhanced hippocampal Dkk-1 levels in C3H control mice as expected, but *reduced* Dkk-1 levels in *doubleridge* mice. This was shown by both immunoblotting (Figure 3A) and immunohistochemistry (Figure 3B). Immunohistochemical analysis also showed that stress reduced β-catenin immunoreactivity in hippocampal pyramidal neurons of C3H mice. Remarkably, β-catenin levels were constitutively low in *doubleridge* mice and did not change in response to stress (Figure 3B). The paradoxical behaviour of Dkk-1 in *doubleridge* mice was confirmed using primary cultures of hippocampal neurons. Basal Dkk-1 levels were higher in cultures prepared from *doubleridge* mice than in cultures prepared from C3H control mice (Figure 3D). Addition of corticosterone (1 µM) enhanced Dkk-1 expression in C3H cultures, but *reduced* Dkk-1 levels in *doubleridge* cultures (Figure 3D).

**Figure 4:**
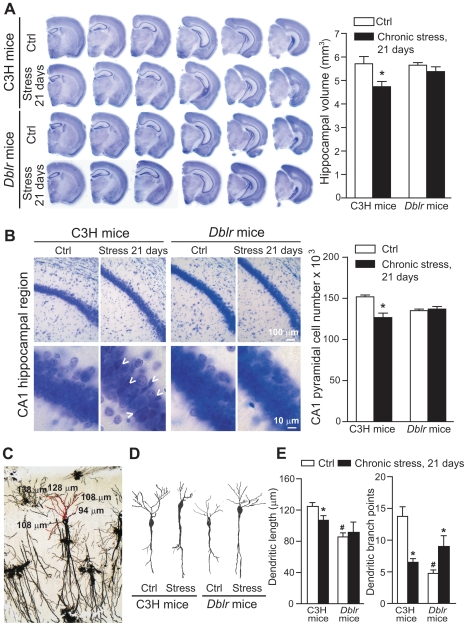
Chronic restraint stress causes hippocampal damage in C3H mice, but not in *doubleridge* mice. Measurements of hippocampal volume in C3H and *doubleridge* (*Dblr*) mice subjected to chronic restraint stress (one a day for 21 consecutive days) is shown in (A). Mice were killed 24 h after the last stress episode. Representative serial Nissl-stained sections showing the entire extension of the hippocampus in unstressed (Ctrl) and stressed CH3 and *doubleridge* mice are shown on the left side. Values are means ± S.E.M. of 8 determinations. *p<0.05 (Student's t test) vs. the respective controls. Stereological counting of Nissl stained neurons are shown in (B). Arrowheads in the left pannels show the presence of piknotic cells. Values are means ± S.E.M. of 8 determinations. *p<0.05 (Student's t test) vs. the respective controls. Golgi staining of CA1 neurons with real measures of dendritic lenght is shown in (C). Camera lucida representative images of CA1 neurons of unstressed or stressed C3H or *Dblr* mice is shown in (D). Mice were subjected to chronic restraint stress as reported in Figure 3. Values shown in the two graphs in (E) are means ± S.E.M. of 8–9 determinations. p<0.05 (One-way ANOVA + Fisher's PLSD) vs. the respective controls (i.e. unstressed mice of the same strain) (*) or vs. unstressed (Ctrl) C3H mice (#).

**Figure 5:**
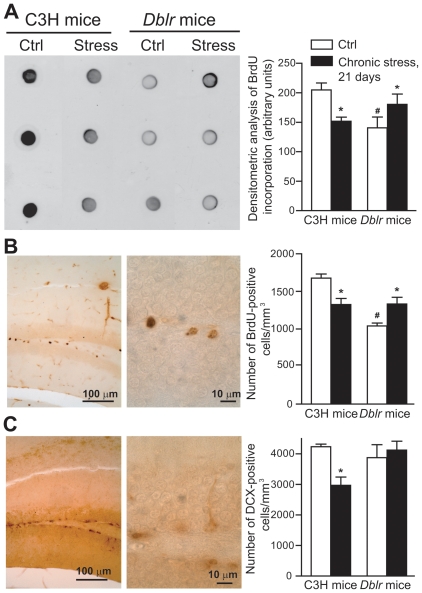
Chronic stress reduces proliferation of neuroprogenitors and neurogenesis in the dentate gyrus of C3H mice, but *enhances* proliferation of neuroprogenitors in *doubleridge* mice. Dot-blot analysis of BrdU incorporation into DNA in hippocampal extracts from unstressed or stressed C3H or *doubleridge* (*Dblr*) mice is shown in (A). Mice were subjected to chronic restraint stress (one a day for 21 consecutive days) and killed 24 h after the last stress episode. A representative dot-blotting is on the left side. Values are means ± S.E.M. of 6–8 determinations. p<0.05 (One-way ANOVA + Fisher's PLSD) vs. the respective controls (unstressed mice) (*) or vs. unstressed (Ctrl) C3H mice (#). BrdU immunostaining is shown in (B) (left side). Stereological values are means ± S.E.M. of 6 determinations. p<0.05 (One-way ANOVA + Fisher's PLSD) vs. the respective controls (unstressed mice) (*) or vs. unstressed (Ctrl) C3H mice (#). Doublecortin (DCX) immunostaining is shown in (C) (left side). Stereological values are means ± S.E.M. of 6 determinations. *p<0.05 (One-way ANOVA + Fisher's PLSD) vs. the respective controls (unstressed mice).

We then examined the Dkk-1 response to chronic stress in the hippocampus of the two strains of mice. C3H mice responded to chronic stress with a long-lasting increase in hippocampal Dkk-1 levels, which peaked in the first 3 days and persisted after 21 days. Thus, C3H mice were similar to CD1 mice in their response to stress. In contrast, in *doubleridge* mice, chronic stress induced an initial reduction in Dkk-1 levels, which then recovered after 1 week (Figure 3C). Using the chronic paradigm of mild restraint stress, we examined the following parameters related to hippocampal damage: (i) hippocampal volume; (ii) stereological counting of CA1 neurons; (iii) dendritic arborization in CA1 neurons; and (iv) proliferation of neuroprogenitors and neurogenesis in the hippocampal dentate gyrus. Hippocampal volume did not differ between C3H and *doubleridge* mice. However, a 21-day restraint stress induced a significant reduction in hippocampal volume in C3H mice, but not in *doubleridge* mice (Figure 4A). We carried out stereological neuronal counting in the CA1 region, where there is evidence of neuronal loss in response to stress/glucocorticoids [48]. Chronic stress caused a 23% reduction in the number of CA1 neurons in C3H mice, but did not cause any significant neuronal loss in *doubleridge* mice (Figure 4B). In C3H mice, chronic stress produced dendritic retraction in CA1 pyramidal neurons, as shown by measurements of dendritic length and dendritic branching (Figure 4C,D,E). Interestingly, *doubleridge* mice showed a reduced dendritic arborisation under basal conditions, and, paradoxically, responded to chronic stress with an *increased* dendritic arborisation (Figure 4D,E). Finally, we measured the effect of chronic stress on proliferation of neuroprogenitor cells and on neurogenesis in the hippocampal dentate gyrus by combining (i) quantitative dot-blot analysis of DNA-containing 5-bromo-2′deoxyuridine (BrdU) in hippocampal extracts [49]; (ii) stereological counting of BrdU-positive cells in the dentate gyrus; and (iii) stereological counting of cells expressing the early neurogenic factor, DCX [50]. In C3H mice, chronic restraint stress significantly reduced BrdU staining as well as the number of DCX-positive cells in the dentate gyrus (Figure 5A,B,C). *Doubleridge* mice showed a lower number of BrdU-positive cells (Figure 5B), but no changes in the number of DCX-positive cells (Figure 5C) in the dentate gyrus, as compared with C3H mice. Once again, stress produced paradoxical effects in *doubleridge* mice, causing an *increased* proliferation of neuroprogenitors, as detected by both dot-blot analysis (Figure 5A) and BrdU immunostaining (Figure 5B).

## Discussion

Our data show for the first time that glucocorticoids induce Dkk-1 in the central nervous system, and suggest that Dkk-1 induction and the ensuing inhibition of the canonical Wnt pathway is a critical component of the molecular cascade leading to hippocampal damage in response to stress. Data obtained with mifepristone in cultured hippocampal neurons suggest that glucocorticoids induce Dkk-1 expression *via* the activation of GRs. This is fully consistent with the evidence that an excessive activation of GRs, as occurs during prolonged exposure to stress, produces neurotoxic effects in the hippocampus, whereas activation of MRs is neuroprotective [18]. The Dkk-1 gene promoter contains at least three glucocorticoid-responsive elements (GREs), and the GR agonist, dexamethasone, enhances Dkk-1 gene expression in human osteoblasts [51]. Thus, induction of Dkk-1 by corticosterone in hippocampal neurons might be mediated by the transcriptional activity of GRs. Alternatively, Dkk-1 induction might be a by-product of the death cascade triggered by GR activation [18]. The Dkk1 gene promoter contains a p53-responsive element, and p53 enhances Dkk-1 expression [52]. In addition, a number of apoptotic agents induce Dkk-1 expression *via* the transcription factor, Jun [53].

Immunohistochemical data obtained in mice exposed to stress or in organotypic hippocampal cultures challenged with corticosterone indicated that Dkk-1 induction was prominent in the CA3 and CA1 hippocampal subfields, which are particularly susceptible to damage induced by stress/glucocorticoids. Accordingly, chronic restraint stress caused dendritic retraction and neuronal loss in the CA1 region (analysis was not extended to the CA3 region). Although stress-induced dendritic atrophy is more often detected in the CA3 region [9]–[11], [13], [14], [54], it also occurs in CA1 neurons [55], where changes in synaptic organization are also observed [56]. All these changes render hippocampal neurons more vulnerable to excitotoxins and other insults (see Introduction and references therein). To examine the relationship between Dkk-1 induction and hippocampal damage, we used transgenic homozygous mice expressing an insertional mutant Dkk1 (*doubleridge*) alleles lacking a transcriptional enhancer. We could not use Dkk1-null mice because they die perinatally due to an incomplete development of structures anterior to the midbrain [47]. In *doubleridge* mice, a transgene insertion in chromosome 19 causes a defective Dkk-1 induction [45], [57], which we have confirmed both *in vitro* and *in vivo*. Unexpectedly, *doubleridge* mice showed higher basal hippocampal Dkk-1 levels associated with dendritic abnormalities in hippocampal neurons as compared to age-matched controls. Thus, perhaps because of the constitutively high expression levels of Dkk-1, *doubleridge* mice showed some phenotypic features shared by wild-type mice exposed to chronic stress. Interestingly, *doubleridge* mice showed a paradoxical response to stress, which caused a *reduction* and not an increase in Dkk-1 levels in hippocampal neurons. In nice agreement with these findings, addition of corticosterone to cultured hippocampal neurons prepared from *doubleridge* mice reduced and did not increase Dkk-1 levels. Chronic stress did not cause any hippocampal damage in *doubleridge* mice, which instead responded to stress with a dendritic remodelling similar to that observed in normal mice during the recovery phase after stress. These data suggest that stress-induced hippocampal damage is causally related to the in Dkk-1 expression (and the inhibition of the Wnt pathway), and that under conditions in which Dkk-1 is reduced rather than increased (i.e. in *doubleridge* mice), chronic stress may have beneficial effects on hippocampal morphology. However, the relationship between Dkk-1 and stress-induced neuronal damage deserves some comments because acute stress did not cause hippocampal damage in control mice in spite of the robust induction of Dkk-1. It is possible that Dkk-1 may be necessary but not sufficient as a death messenger, and other events induced by chronic stress, such as the reduction of brain-derived neurotrophic factor [1], enable the development of hippocampal damage. We also used *doubleridge* mice to examine whether induction of Dkk-1 was instrumental for the reduction of hippocampal neurogenesis associated with chronic stress. Activation of the canonical Wnt pathway supports self-renewal and neuronal fate commitment of neural stem/progenitor cells of the adult hippocampal dentate gyrus [28], [58]–[60]. The regulation of adult neurogenesis by Dkk-1 has never been studied. Using the HB1.F3 line of immortalized neural stem cells, Ahn et al. [61] have found that olig2-induced cell differentiation into astrocytes, oligodendrocytes, and neurons required the induction of Dkk-1 and the resulting inhibition of the canonical Wnt pathway. In C3H mice, chronic stress reduced BrdU incorporation in hippocampal neuroprogenitors, as well as the number of cells expressing DCX, an early marker of neuronal development in the adult hippocampus that is expressed by type-2b progenitor cells up to the stage of immature granule cells [50]. *Doubleridge* mice showed a reduced BrdU incorporation into hippocampal neuroprogenitors, which, again, might reflect the elevated basal Dkk-1 levels found in the hippocampus of these mice. Interestingly, chronic stress enhanced BrdU incorporation in *doubleridge* mice, an effect commonly seen with antidepressants in experimental animals and humans [60], [62]–[64]. However, it should be highlighted that the relationship between the mood-improving action of antidepressants and the increase in hippocampal neurogenesis is still matter of debate [65], [66].

We conclude that a strong relation exists between Dkk-1 induction and hippocampal damage in response to stress. This suggests that drugs that rescue the canonical Wnt pathway may be effective in preventing hippocampal damage associated with stress-related disorders, including major depression. Lithium ions and valproate activate the Wnt pathway acting as weak inhibitors of GSK3β [67], [68]. Interestingly, lithium ions are known to prevent dendritic abnormalities in the hippocampus and amygdala [69], [70], and to reverse the reduction in the proliferation of adult dentate-gyrus derived neural precursor cells induced by glucocorticoids [71]. However, lithium ions and valproate have multiple mechanisms of action and their use is limited by serious adverse effects. Dkk-1 antagonists or selective GSK3β inhibitors may be better candidates as neuroprotective agents in stress-related disorders. Finally, data shown in *doubleridge* mice raise the intriguing possibility that chronic stress may produce positive effects on hippocampal neuroplasticity in the presence of agents that limit Dkk-1 induction or rescue the Wnt pathway. This attractive hypothesis warrants further investigation.

## Materials and Methods

### In vivo experiments

#### Animals and treatments

Experiments were performed following the Guidelines for Animal Care and Use of the National Institutes of Health. The study was approved by Italian Ministry of Health (permit number 432007/A).

Male CD1 mice, C3H mice (Charles River, Calco, Italy) or mutant mice homozygous for an insertional mutant allele of Dkk1 gene lacking a transcriptional enhancer (*doubleridge* mice) (kindly provided by Miriam H. Meisler, Ann Arbor, University of Michigan, USA), 10 week-old, weighing 22–24 g were used. All mice were kept under environmentally controlled conditions (room temperature  = 22°C, humidity  = 40%) on a 12-h light/dark cycle with food and water *ad libitum*. For the induction of restraint stress, mice were placed for 3 h in plexiglas restrainers (length: 5 cm; width: 5 cm). The restraint device contained a 0.4 cm air hole and allowed mice to stretch their legs but not to move within the tube. Experimental manipulations were performed between 9.30 and 11.30 a.m. Mice were subjected either to single or repeated restraint stress. In the latter case, mice were placed in the restrainers 3 h per day for 21 consecutive days. Mice subjected to acute stress were killed 1, 3 or 7 days after stress. Mice subjected to chronic stress were killed 24 h after the last restraint episode at days 1, 3, 7, 14 and 21. Some groups of CD1 mice that had not been exposed to stress received an acute s.c. injection of corticosterone (1 mg/kg) dissolved in 30% DMSO/70% saline. Control mice received an equal volume of vehicle. All mice were handled gently for 5 consecutive days prior to the injections to minimize stress associated with injections. Mice were killed 24 h after injection. Additional groups of CD1 mice were adrenalectomized under pentobarbital anaesthesia, as described [38]. After surgery, all animals were given free access to 0.9% NaCl in addition to normal drinking water. Adrenalectomized mice were subjected to an acute s.c. injection of corticosterone (1 mg/kg) dissolved in 30% DMSO/70% saline or to an acute episode of restraint stress as described above, and killed 24 h later.

#### Dot-blot analysis of BrdU incorporation

Mice were injected with BrdU (Sigma; 3 i.p. injections of 50 mg/kg, with 2 h of interval, cumulative dose: 150 mg/kg,) and killed 24 h later. Mice were anesthetized with chloralium hydrate (320 mg/kg, i.p.), and perfused with 30 ml of saline. Hippocampi were dissected, minced quickly with a razor blade, and frozen at 80°C. Genomic DNA was extracted from hippocampal tissues by a DNA isolation buffer (50 mM Tris HCl, pH 8.0, 100 mM NaCl, 100 mM EDTA, 1% SDS, 1 mg/ml proteinase K) overnight at 56°C. Genomic DNA was precipitated by centrifugation at 13,000 *g*, washed twice with 100% ethanol, and suspended in Tris-EDTA buffer (10 mM Tris HCl, pH 8.0, 1 mM EDTA). To eliminate RNA, the DNA samples were incubated for 1 h at 37°C with ribonuclease A (33 µg/ml, Sigma). Purified DNA was quantitated by spectrophotometry and 50 µl were single stranded by incubation with 10 µl of 0.4 N NaOH for 30 min at room temperature and kept on ice to prevent annealing. The DNA solution was placed on ice and neutralized by an equal volume of 1 M Tris HCl (pH 6.8). The single-stranded neutralized DNA (50 ng/5 µl) was dot-blotted onto a nitrocellulose membrane (Bio-Rad, Hercules, CA).

To visualize the BrdU signal, membranes were incubated with mouse anti-BrdU monoclonal antibodies (1∶100, Becton Dickinson) in a buffer containing 20 mM Tris HCl, pH 7.6, 136 mM NaCl, and 0.05% Tween 20 (TBS-T) containing 1% non-fat milk overnight at 4°C. After being washed with TBS-T, membranes were incubated with a horseradish peroxidase-conjugated anti-mouse IgG antibody (1∶5,000; EMD Biosciences) for 1 h at room temperature. Membranes were washed with TBS-T, analyzed by enhanced chemiluminescence (GE Helthcare), and exposed to autoradiography film (Daigger, Vernon Hills, IL). Intensity of each BrdU signal was quantitated by densitometric analysis using Kodak one-dimensional software.

#### Immunohistochemistry and Nissl staining

Mice were anesthetized with chloralium hydrate (320 mg/kg, i.p.) and transcardially perfused with 4% paraformaldehyde (PFA) in 0.1 M phosphate buffer, pH 8. Brains were removed, post-fixed overnight in 4% PFA, and then transferred in 30% sucrose for cryoprotection. Serial 30 µm coronal sections were obtained from the whole rostrocaudal extent of the hippocampus and processed for Dkk-1 and β-catenin immunostaining using the following primary antibodies: goat polyclonal Dkk-1 (1∶10; R&D System Inc., Minneapolis, MN) and rabbit polyclonal anti-β-catenin (1∶10; Santa Cruz Biotechnology Inc., Santa Cruz, CA); sections were then exposed for 1 h to secondary biotinylated anti-goat or anti-rabbit antibodies (1∶200; Vector Laboratories, Burlingame, CA). 3,3-Diaminobenzidine tetrachloride was used for detection (ABC Elite kit; Vector Laboratories). Control staining was performed without the primary antibodies.

For (i) immunohistochemical analysis of cells expressing BrdU (in mice injected with BrdU - see above -), (ii) immunohistochemical analysis of cells expressing doublecortin (DCX), and (iii) histological analysis, brains were fixed in Carnoi (ethanol:acetic acid:chloroform, 6∶1∶3), embedded in paraffin and sectioned at 30 µm. Deparaffinized sections were soaked in 3% hydrogen peroxide to block endogenous peroxidase activity. Sections were treated with 10 mM, pH 6.0, citrate buffer, and heated in a microwave for 10 min for antigen retrieval. The slides were allowed to cool for 20 min in the same solution at room temperature and then washed in TBS. The sections were incubated overnight with mouse monoclonal anti-BrdU (1∶20, Becton Dickinson) or rabbit polyclonal anti-DCX (1∶50, Santa Cruz Biotechnology Inc.), and then for 1 h with secondary biotinylated anti-mouse or anti-rabbit antibodies (1∶200; Vector Laboratories, Burlingame, CA). 3,3-Diaminobenzidine tetrachloride was used for detection (ABC Elite kit; Vector Laboratories). Control staining was performed without the primary antibodies.

Deparaffinized sections were also processed for staining with thionin (Nissl staining). After rinses in dH_2_O, sections were incubated for 8 min in thionin. The number of surviving neurons in the pyramidal cell layer was counted by a stereological method (see below). The mean hippocampal volume was determined by quantitative light microscopical methods using the Cavalieri's method [39], [40]. Rostrocaudal sections from the entire hippocampus of each animal were taken every 150 µm and the volume was calculated by using the following formula: V = Σ(A)i*TS*n where **Ai** is the area measured outlining the regions of interest on section i^th^ at 2.5× magnification, **TS** the section thickness (30 µm), and **n** the number of sections through the levels.

#### Stereological counting

Stereological cell counting was performed using the ImagePro Plus 6.2 software coupled to a Axio Imager M1 microscope. The optical fractionator technique (adapted to 30 µm thick sections) was used to obtain estimates of total cells numbers within the hippocampus [41], [42]. A systematically sampled series of sections every 150 µm spanning the entire extent of the hippocampus was selected for quantification. The number of BrdU or DCX-positive cells was quantified on both hippocampi (n = 5 mice for each experimental group). During quantification, the regions of interest were outlined on the section at 2.5× magnification. After outlining the regions of interest, a sampling grid of known dimensions (75×75 µm) was positioned over each area and counting was carried out using a 100X oil immersion lens [43].

#### Assessment of dendritic arborisation in hippocampal pyramidal cells

Mice were anesthetized with chloralium hydrate (320 mg/kg, i.p.) and transcardially perfused with 4% PFA in 0.1 M phosphate buffer, pH 7.4. Brains were removed, post-fixed overnight in 4% PFA, and then transferred in 30% sucrose for cryoprotection. Brains were quickly frozen in 2-methylbutane, and processed according to the commercial rapid Golgi staining kit (FD Neurotechnologies Consulting & Services, Ellicott City, MD). In brief, whole brains were treated for silver impregnation for 2 weeks. Sixty µm brain sections, obtained on a cryostat, were stained and mounted in resinous medium. Dendritic length and branch points (number of dendritic bifurcations) were quantified using the ImagePro Plus 6.2 software coupled to an Axio Imager M1 microscope. Briefly, this equipment allows to perform a 3 dimensional reconstruction of the neuron by merging images acquired on different focal planes (every 5 µm). We superimposed a manual line from the neuronal soma up to the single dendrite tip.

### In vitro experiments

#### Organotypic hippocampal slice cultures

Postnatal day 6–7 (P6/7) mouse organotypic hippocampal slice cultures were prepared as described previously [44]. Briefly, hippocampi were dissected in ice-cold dissecting medium (50% Minimal Essential Media (MEM) with no bicarbonate, 50% calcium and magnesium-free Hank's Balanced Salt Solution (HBSS), 7.5 mM D-glucose, 20 mM HEPES, pH 7.15). Hippocampi were isolated and then sectioned coronally at 300 µm thickness with a mechanical tissue chopper. The slices were separated and transferred to sterile, porous membrane units (0.4 µm, Millicell-CM, Millipore, Temecula, CA), five per insert, containing 1 ml of culturing medium in each dish (50% MEM with Earle's salt and L-glutamine, 25% HBSS, 25% horse serum with 6.5 mg/L D-glucose, 20 mM HEPES and 50 U/ml streptomycin-penicillin). Culture medium was replaced every 2 days, and slices were kept at 37°C in 5% CO_2_ for 9 days before experiments. Corticosterone (10 nM, 100 nM or 1 µM) was applied for 24 h. At the end of treatment, slices were fixed in 4% PFA. After rinsing, slice were blocked with 5% normal goat serum diluted in PBS with 1% Triton X-100 for 2 h. Slices were incubated overnight with the primary goat polyclonal anti-Dkk-1 antibody (1∶10; R&D System). After rinsing, slices were incubated with a secondary biotinylated anti-goat (1∶200; Vector Laboratories, Burlingame, CA). 3,3-Diaminobenzidine tetrachloride was used for detection (ABC Elite kit; Vector Laboratories). Alternatively, fluorescence immunohistochemistry was performed incubating brain slices overnight with goat polyclonal anti-Dkk-1 (1∶10; R&D System) and then for 5 min with propidium iodide (1∶10).

#### Preparation of primary cultures of hippocampal neurons

Cultured neurons were prepared from the hippocampus of E14-16 C3H and *doubleridge* mice. In brief, dissected hippocampi were triturated with a fire-polished Pasteur pipette to dissociate the tissue into single cells. After centrifugation (1,000 *g*, 7 min), the cell pellet was resuspended in Neurobasal Medium supplemented with B27, and 500 μM L-glutamine and plated at a density of 4×10^5^ to 7×10^5^ cells/ml in culture dishes coated with poly-D-lysine (molecular weight 70,000–150,000, 0.1 mg/ml; Sigma, St. Louis, MO).

Cultured neurons were also prepared from the hippocampus of Sprague-Dawley rat pups (P1-P2) (Charles River). Briefly, hippocampi were dissected in Ca^2+^/Mg^2+^ free buffer and mechanically dissociated. Dissected hippocampi were then triturated with a fire-polished Pasteur pipette to dissociate the tissue into single cells. After centrifugation (1,500 *g*, 6 min), the cell pellet was resuspended in Neurobasal Medium supplemented with B27, and 500 μM L-glutamine and plated at a density of 4×10^5^ to 7×10^5^ cells/ml in culture dishes precoated with 0.1 mg/ml poly-D-lysine.

All cultures were grown in a humidified atmosphere containing 5% CO_2_ at 37°C. At 10 DIV, cultures were incubated with corticosterone (0.01–1 µM for 16–24 h) in the presence or absence of spironolactone (10 µM) and mifepristone (10 µM); afterwards, cells were washed three times with ice-cold PBS and lysed with lysis buffer (20 mM Tris, 1 mM EDTA, 1% Triton X-100, 10% glycerol, 1 mM phenylmethylsulfonyl fluoride, 1 µg/ml aprotinin, 1 µg/ml pepstatin and 1 µg/ml leupeptin) for 15 min and used for Western blot analysis.

#### Western blot analysis

Western blot analysis of Dkk-1 was performed in protein extracts of primary hippocampal cultures or in protein lysates obtained from mouse brain regions. Blots were incubated overnight at 4°C with the goat polyclonal Dkk-1 primary antibody (2 µg/ml, R&D System). Blots for mouse monoclonal β-actin (Sigma) were incubated for 1 h at room temperature (1∶60,000). Filters were washed three times with TTBS buffer (100 mM Tris-HCl, 0.9% NaCl, and 0.1% Tween 20, pH 7.4) and then incubated for 1 h with secondary peroxidase-coupled antibodies (anti-goat 1∶2,000 or anti-mouse, 1∶7,000; EMD Biosciences, Darmstadt, Germany). Immunostaining was revealed by enhanced-chemiluminescence luminosity (GE Healthcare, Buckingamshire, UK).
